# The New York Institute of Stomatology

**Published:** 1902-07

**Authors:** 

**Affiliations:** The New York Institute of Stomatology


### Reports of Society Meetings.

### THE NEW YORK INSTITUTE OF STOMATOLOGY.

A MEETING of the Institute was held at the office of Dr. W. E. Hoag, 8 East Forty-third Street, New York, on March 4, 1902, the President, Dr. Howe, in the chair.

The minutes of the last meeting were read and approved. The chairman announced that a paper by Dr. F. Milton Smith, on the subject of preparing gold tips for teeth would be substituted for the paper which was to have been presented on replacing broken porcelains on bridge-work, etc.

*Dr. Smith.*—What I have to present can hardly be dignified by the name of a paper. It is simply a detailed description of this process and was prepared in compliance with a request of the editor of the INTERNATIONAL DENTAL JOURNAL for short papers of a practical nature.

(For Dr. Smith's paper, see page 483.)

### DISCUSSION.

*Dr. G. A. Wilson.*—I wish to state that six or eight weeks ago Dr. Smith put two such tips on my front teeth. I have had considerable experience, and I have found them to be satisfactory pieces of work. I have had one or two cases in the last two or three years, and I would like to add one or two suggestions. Instead of pure gold as a starter, I use gold with two per cent. of platinum. It sets down better than pure gold, and instead of two pins I use three. Like three legs to a stool, it holds the tip in place better even if one pin is very shallow. Instead of pure platinum for a pin, I use platinum-iridium wire, which is much stiffer and can be used much smaller.

*Dr. I. F. Wardwell.*—When the platinum pin was spoken of I remembered that Dr. Smith described that method to me over two years ago, and I was very glad to make use of what he had told me. I find the platinum-iridium pin to be a great deal smaller and stiffer. I can indorse this kind of work.

*Dr. Chas. O. Kimball.*—In the case of a canine tooth worn down, involving a large filling on the side, as well as the grinding surface, the gentleman having a very heavy bite, in addition to the two pins I placed a third pin up on the side of the tooth at the bottom of the lateral cavity. I cut the hole so the pin would slide up from below, so preventing the filling from spreading out. If the cavity had extended on both sides this would not have been necessary. It worked admirably.

*Dr. F. Milton Smith.*—I want to disclaim any originality for this method. To Dr. Dwight Smith I am as much indebted as to any other, unless it be to Dr. Littig. But in order that those who are not familiar with this work may know the details I prepared this paper.

Dr. Justin de Lisle presented a paper entitled "Some Popular Errors about Microbes."

(For Dr. de Lisle's paper, see page 469.)

DISCUSSION.

*Dr. S. A. Hopkins.*—I cannot let this opportunity go by without saying a word. When I heard that Dr. de Lisle was going to read a paper I happened to be in the Bacteriological Laboratory of the Harvard Medical School. I called Dr. Ernst's attention to the notice I had received, and by way of coincidence he picked up a manuscript with Dr. de Lisle's name upon it, which he said was for publication in the *Journal of Medical Research.* I felt that anybody who was a contributor to the *Journal of Medical Research* was worth listening to, and I have been very much interested and am very glad that I came on. However, I do not altogether agree with the conclusions of the essayist. In the first place, antitoxins, with the exception of the antitoxin for diphtheria, are not, as a rule, sufficiently certain in their results to be trifled with. I do not think any physician would be justified in injecting the antitoxin of tetanus because a patient had received a slight wound and there was a very remote possibility of the tetanus bacillus entering that wound. As to the bacteria of the mouth, it is pretty difficult to say how many there are. Of course, nearly every form of bacteria gets into the mouth at one time or another. I have isolated over forty different varieties which I have found in the mouth, but I should say that comparatively few, certainly not more than quarter of that number, would be looked upon as mouth bacteria only.

As to the action of saliva in exciting the phagocytes to action, I think, from the result of a few experiments that I have made, that sterilized saliva has very little power to stimulate the production of these protecting phagocytes. Unsterilized saliva, however, does not seem to produce this action, which would lead one to believe that it might be not the saliva itself, but some organism in the saliva, which brought about this action. In regard to the sterilization of instruments I have only this to say: It is, of course, impossible to work under strictly aseptic conditions in the mouth, because the mouth itself teems with bacteria, but I think every patient who comes into the office of any dentist is entitled to protection from the patient who has been operated on before him, and I do not think any suggestion of carelessness such as has been intimated in the essay that we have listened to should be entertained for a moment.

*Dr. H. W. Gillett.*—I have been exceedingly interested in the point of view Dr. de Lisle has taken, but there are certain points that appeal to me from the stand-point of a practitioner. Dr. de Lisle's statements have dealt with the assumption that the operative dentist has only to do with the hard dentine. Practically it is something very different, and I should be very careful to protect my patient from the one who has preceded him where pulpless teeth with open foramina and gum-tissue are concerned.

*Dr. R. H. M. Dawbarn.*—I hope the Lord will give me the power, Mr. President, to sit down within the course of the next five or ten minutes, because enough points have been raised that deserve discussion to keep me busy for the next week. In the first place I wish to say to you all how much I esteem Dr. de Lisle personally, and when I am through I hope he will still shake me by the hand. I have never in my life seen a more striking instance of the danger of a theoretical man trespassing upon practical grounds. Here is a man who spends his life in the laboratory, not seeing disease at all, and draws conclusions from a laboratory study which are absolutely dangerous to human life.

If his statements had reference to the extremes of general surgeons as to *antisepsis,* as regards its being overdone, I would not take issue with him; but he is absolutely wrong if he thinks surgeons, whose business it is to teach, are in the least doubt about the wisdom of absolute *asepsis* before surgical operations. Asepsis simply means surgical cleanliness, and there will never be a point upon which surgeons are more in unison than upon this. It is impossible to sterilize the skin as it should be done in a few minutes' time, and it is a fact that if you scrape the hands you stir up some of the deeper layers of microbes, and one should avoid this. For this reason, and in order to avoid microbes of all kinds, the majority of surgeons now use boiled rubber gloves, first, however, making the hands as aseptic as possible, to avoid infecting the wound if a glove be torn or cut during the operation. Rubber gloves have come to stay; they have logic on their side; and it behooves the minority to defend themselves and not the majority.

Early in Dr. de Lisle's address he said in effect that a great many surgeons are contemplating using tetanic serum as a preventive measure. I do not know of a surgeon in the New York Surgical Society who has the least idea of doing so. I for one should consider it reckless. There is in prevention of tetanus a better means and a much simpler one. You all know that the bacillus tetani is anaërobic; it cannot multiply in the presence of oxygen; and consequently it can be prevented from multiplying by exposing the infected surfaces to free access of air by drainage-tubes or free use of gauze drains. He mentions that surgeons contemplate the injecting of the serums of the different microbes before an operation, in order to do away with all this antisepsis. The trouble is that in most cases we have a very mixed infection, and we can never be sure which ones among many microbes there will be, and consequently we do not know what serum to inject. I do not believe this theory ever emanated from the mind of a practical surgeon.

Regarding intestinal microbes, there is not any question but that they are beneficial, breaking up the substances in the alimentary canal and assisting assimilation and elimination. In regard to the animals fed upon perfectly sterile food and breathing sterile air, they undoubtedly died from this cause. No doubt some microbes do have the power of helping us in certain ways. Most of the microbes in the alimentary canal are such as live upon dead animal tissue and are not of the variety that attack living animal tissue.

Regarding lupus and its slow development in the skin, I do not think the doctor has put his finger upon the true cause why tuberculosis does not go on as rapidly in the skin as tubercular processes more deeply placed. It is because, of all the pathogenic bacteria, that of tuberculosis is the one most easily affected by even slight changes in temperature; and in the skin the temperature is enough cooler than that within the lungs, for example, to retard the growth of tubercle bacilli. For this reason, in the treatment of tuberculosis of the lungs, ice-bags externally, or, upon the other hand, breathing extremely hot air, have been sometimes used. It was not much of a success, however.

Regarding chemiotaxis, I have no doubt but later some medicinal substance will be found to increase it for the benefit of humanity.

The speaker alluded to the baking of instruments. Surgeons do not bake their instruments, they boil them. I do not think that in a thousand years from now we will have improved our method of sterilizing instruments, because it is perfect. In five minutes they can be thoroughly sterilized, and without expense, in boiling water with one to five per cent. of washing (not baking) soda added, to prevent rusting. I am informed by an eminent metallurgical chemist, Mr. Le Boutillier, that it is a mistake to suppose that a temperature of 212° F. can in any way injure the temper of steel. Since that time I have always boiled all my instruments together, first wrapping the knife blades in a little gauze to prevent accidental dulling by contact with other tools.

I was particularly sorry at the intimation that we should not use the utmost care in sterilizing the instruments used in dentistry. There is too little care used, as a rule, even at the present time. My colleague, Dr. Wyeth, president of the American Medical Association, remarked to me some months ago that he was almost afraid to go to a dentist because he did not know whom he could trust in the matter of cleansing instruments. I hastened to assure him that I knew of a number of excellent dentists who were very careful about sterilizing their instruments.

I wonder how many of you gentlemen realize the large proportion of syphilis there is in the community. Your patients and my patients will commonly simply lie to us with regard to it if, having it, they are asked. Human nature is much the same here as in Paris. One man in ten in the public hospitals there is syphilitic, to quote Dr. Prince A. Morrow. Of course, this percentage is not so large in the better portion of the community, but, notwithstanding, it is so high that the greatest care needs to be taken to avoid carrying the infection from one patient to another. Dr. de Lisle has stated that the interval of a few minutes which must elapse in carrying the infection from one person to another is sufficient to destroy the poison; but I think none of us would upon our own persons dare risk infection from instruments used even days before upon a known syphilitic unless these tools had been sterilized in the interval. The admitted fact that none of us knows of an instance of syphilis conveyed by dental instruments proves nothing at all. Nothing could be more nearly impossible to prove; and yet syphilis from use of forks, spoons, and drinking-vessels in common is far from rare; hence I am forced to believe that it is at times conveyed by carelessly cleansed dental implements.

In conclusion, I think, gentlemen, that we who are teachers, and I am sure Dr. de Lisle properly classes himself in that category, should be extremely careful not to convey anything which can be taken as an excuse for relaxing aseptic measures, even jocularly. I am reminded of the advice of the old orthodox preacher to the young minister: "My boy, never dilute your damnation. Your congregation will do that for you."

*Dr. E. A. Bogue.*—Superstition has been defined as the fear of the unknown. I have many times thought how much superstition there is in the community regarding bacterial life. I could not help having this definition come to me as I heard Dr. de Lisle's paper. I did not understand him in the least to argue against proper precautions nor the utmost care, but I did understand him to say just what Dr. Dawbarn has said, that aseptic surgery is clean surgery. I am reminded of something I read a little while ago from Dr. Chapin regarding filth diseases. Dr. Chapin says, "As soon as the germ theory of disease ceased to be a mere theory, and the true facts in regard to the etiology of infectious diseases began to be known, and bacteriology gave us exact knowledge of the life history of the minute organisms which are the cause, the erroneous generalizations of the filth theory became apparent. We can now, to a large extent, discriminate between filth that is dangerous and filth that is not." It is not very many months ago that a gentleman said in the presence of this Institute that instruments used in the case of a syphilitic patient should never be used again. I have understood Dr. de Lisle to say that he has seen, with his own eyes, under the microscope, the death of these syphilitic germs; that while the germ was a living one when he placed it upon the slide, it died before he stopped looking. If this is so, it need not teach us that we can be careless in our measures for cleanliness, but we can take a great deal of comfort to ourselves in knowing that, in view of the numerous carelessnesses that are sure to happen right along, we may be assured that we are not conveying this dread disease on our instruments. Referring again to Dr. Chapin's remarks, cholera and typhoid fever are both propagated by bacteria that are taken into the mouth, and the tubercle bacilli are carried around by all sorts of breezes. Yellow and malarial fevers are conveyed by mosquitoes of two varieties,—malarial by a mosquito that lives in clean water, and yellow fever by one that lives in dirty water, and yet we find men who work in sewers are not seriously affected by the filth they encounter.

For my part I thank Dr. de Lisle for relieving my mind regarding some of these infectious diseases, but, nevertheless, I shall keep my instruments just as clean as heretofore, while I shall not have the superstitious fear that leads to an over-solicitude that may do as much harm as it seeks to avoid.

*Dr. J. Morgan Howe.*—I think we are under great obligation to Dr. de Lisle for the paper he has given us. I do not sympathize with that old minister's advice with regard to the strength of the damnation that he would have preached, because his hearers would discount it. I think the simple truth will be always safe to rest on. I think the facts that Dr. de Lisle has given us will do us good. I shall, however, continue my practice of sterilizing instruments, because even remote chances of infection should be eliminated. I do not think Dr. de Lisle means in the least to question the advisability of using all the precautions that are required to prevent our instruments, materials, and everything we use from any possibility of communicating disease from one patient to another.

*Dr. de Lisle.*—I have very little to say in conclusion except that I thank you all for your kind and just criticism. There are, however, one or two words that I would say. One of the gentlemen has stated that "where there is a possibility of tetanus he flushes the wound with oxygen;" "that the bacillus does not live in oxygen." However, it does live as a spore, and as a spore it gets into the wound. The spore will not germinate in the presence of oxygen, but after the wound has healed, and because it is impossible to flush the cicatrix with oxygen, this inhibiting action is lost and the spore becomes an active bacillus.

*Dr. Dawbarn.*—And would you advocate the injection of an antitoxin?

*Dr. de Lisle.*—Most assuredly. One of the affinities of the toxine of tetanus is for nerve-tissue. The toxine of tetanus fixes itself upon nerve-tissue and follows it up through the spine to the brain, and the consequence is that we do not know that we are in the presence of tetanus until the patient is beyond hope, because the first symptom is the setting of the jaws, and then it is too late, because the toxine has already arrived at the brain. A cure may be effected by opening the skull and injecting the serum directly into the brain. The advantages can be readily seen of injecting the serum before the spore has become a bacillus. I thank you very much, gentlemen.

*Dr. F. Milton Smith.*—Dr. Geo. S. Allan, at our meeting two months ago, read a paper entitled "Extension for Prevention," in which he spoke of Dr. Black's instruments. It has been suggested that some of our Eastern men are very much behindhand, as much as forty years, I think, because they presumably had never heard of Dr. Black's instruments. Dr. Allan has secured a full set of Dr. Black's instruments. They are here for your inspection.

Adjourned.

FRED. L. BOGUE, M.D., D.D.S.,
*Editor The New York Institute of Stomatology.*

